# Structure and host specificity of *Staphylococcus epidermidis* bacteriophage Andhra

**DOI:** 10.1126/sciadv.ade0459

**Published:** 2022-11-30

**Authors:** N’Toia C. Hawkins, James L. Kizziah, Asma Hatoum-Aslan, Terje Dokland

**Affiliations:** ^1^ Department of Microbiology, University of Alabama at Birmingham, Birmingham, AL 35294, USA.; ^2^ Department of Microbiology, University of Illinois Urbana-Champaign, Urbana, IL 61801, USA.

## Abstract

*Staphylococcus epidermidis* is an opportunistic pathogen of the human skin, often associated with infections of implanted medical devices. Staphylococcal picoviruses are a group of strictly lytic, short-tailed bacteriophages with compact genomes that are attractive candidates for therapeutic use. Here, we report the structure of the complete virion of *S. epidermidis*–infecting phage Andhra, determined using high-resolution cryo–electron microscopy, allowing atomic modeling of 11 capsid and tail proteins. The capsid is a *T* = 4 icosahedron containing a unique stabilizing capsid lining protein. The tail includes 12 trimers of a unique receptor binding protein (RBP), a lytic protein that also serves to anchor the RBPs to the tail stem, and a hexameric tail knob that acts as a gatekeeper for DNA ejection. Using structure prediction with AlphaFold, we identified the two proteins that comprise the tail tip heterooctamer. Our findings elucidate critical features for virion assembly, host recognition, and penetration.

## Teaser

The structure of *S. epidermidis* phage Andhra reveals host determinants and highlights the therapeutic potential of picoviruses.

## INTRODUCTION

Staphylococci are dominant constituents of the skin microbiome that play critical roles in health and disease. Of the more than 40 skin-associated *Staphylococcus* species (*1*), *S. aureus* and *S. epidermidis* have the greatest pathogenic potential. *S. aureus* is a major cause of a wide range of diseases, from skin and soft tissue infections to lethal sepsis and bacteremia (*2*–*5*). Although *S. epidermidis* is largely regarded as a beneficial commensal organism, it is also a leading cause of infections of indwelling medical devices (*6*–*10*).

The emergence of antibiotic resistant strains of *S. aureus* (*11*) and *S. epidermidis* (*12*, *13*), combined with a paucity of new antibiotics in the development pipeline (*14*, *15*), has led to a renewed interest in the therapeutic use of bacterial viruses, or bacteriophages, to eradicate staphylococcal infections (*16*–*18*). However, many challenges impede the routine use of phage therapy, including the narrow host range of phages, rapid evolution of resistance, and concerns about uncharacterized phage genes and potential for genetic mobilization (*19*–*21*).

**Fig. 1.**
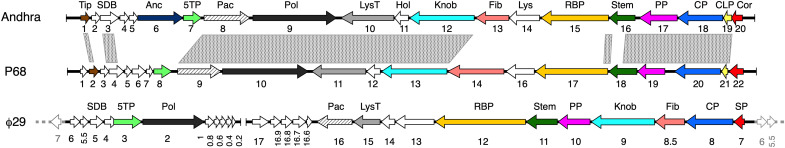
Comparison of Andhra, P68, and ϕ29 genomes. ORFs are numbered in each genome, and relevant genes are labeled. Genes encoding structural proteins are color coded: Tail tip protein (Tip), brown; RBP anchor (Anc), dark blue; terminal protein (5TP), light green; tail tip lysin (LysT), gray; tail knob protein (Knob), cyan; head fiber (Fib), salmon; RBP, gold; tail stem/collar protein (Stem), green; portal protein (PP), magenta; major capsid protein (CP), blue; capsid lining protein (CLP), yellow; core (Cor)/scaffolding (SP) protein, red. Nonstructural proteins include the packaging ATPase (Pac), hatched; the DNA polymerase (Pol), black; and the single-stranded DNA binding protein (SDB), holin (Hol) and endolysin (Lys), white. The stippled areas between the Andhra and P68 genomes indicate regions of >70% nucleotide identity, calculated in BLASTN (*29*). The ϕ29 genome was split between ORFs 7 and 6, and the right end was moved to the left side (indicated by the duplicated ORFs in gray) to facilitate a more direct comparison to Andhra and P68.

The picoviruses are a subgroup of the family *Podoviridae* (podoviruses) of short-tailed phages (*22*) that includes the recently designated families *Rountreeviridae* and *Salasmaviridae* (*23*, *24*). The most well-described member of this group is *Bacillus* phage ϕ29 (family *Salasmaviridae*) (*25*, *26*), but several members that infect *S. aureus* are also known, including 44AHJD and P68 (family *Rountreeviridae*) (*27*). Picoviruses are strictly lytic, with genomes of only ≈18 to 20 kilo–base pairs (Fig. 1) and use a unique replication and genome packaging strategy based on monomeric genomes with terminal proteins covalently bound to their 5′ ends (*28*). These features make picoviruses attractive for therapeutic applications. However, picoviruses are rare in phage collections and thus have remained relatively understudied and underused.

We recently described several new staphylococcal picoviruses, including the *S. epidermidis*–infecting phage Andhra (*29*, *30*). These phages are related to other staphylococcal picoviruses in the family Rountreeviridae (*23*) and share a similar genome organization, with high conservation of core genes involved in DNA replication, packaging, and virion structure (Fig. 1). A comparison of the genomes of phages Andhra and P68 also revealed regions of divergence in Andhra open reading frames (ORFs) 2 to 7 and ORFs 13 to 15 (*29*), which likely reflect host-specific differences.

A major determinant of phage host specificity is the bacterial surface structures that serve as phage receptors. In the case of staphylococci and other Gram-positive bacteria, the primary receptor is often wall teichoic acid (WTA), a variable polymer present on the surface of most Gram-positive cells (*31*, *32*). Phages bind to these surface structures using a variety of fibers or receptor binding proteins (RBPs) present on their virions. However, the structural basis for host specificity between phages that infect *S. aureus* and *S. epidermidis* is still poorly understood.

Here, we have determined the high-resolution structure of the *S. epidermidis*–infecting phage Andhra virion using cryo–electron microscopy (cryo-EM), allowing atomic models to be built for all or part of 11 different structural proteins in the capsid and tail, including the RBPs and other proteins involved in penetration of the host cell wall. This is the first structure of a *S. epidermidis*–infecting phage and exposes the structure of a picovirus in unprecedented detail. Comparison to P68 allows host-related differences to be examined. Andhra exhibits several unique features compared to P68, including a distinct RBP and a unique lytic protein that also serves to anchor the RBPs to the tail. Our data indicate that the structure of the RBPs and the lytic proteins reflect the type of WTA and cell wall composition of the host. Unlike previous picovirus structures, we were able to use focused reconstruction to resolve the knob protein hexamer, which acts as a gatekeeper for DNA ejection, and the tail tip, which is formed by a heterooctamer of two proteins. The tail tip lysins insert into the knob and stabilize the internal loops that hold back the terminal protein and the DNA, thus coupling cell wall penetration to DNA release. Our study has also shown how structure prediction with AlphaFold (*33*) can be used in combination with atomic modeling in moderate resolution maps obtained by cryo-EM to identify novel structural proteins and correct for misannotations in the genome data bank.

## RESULTS

### Overall structure and protein composition of the Andhra virion

Purified phage Andhra was analyzed by mass spectrometry (MS). The measured masses were compared to the predicted ORFs from the Andhra genomic sequence (Fig. 1). Gene products corresponding to 19 of the 20 ORFs were identified in the sample, including several expected capsid and tail proteins (Table 1). Some of the detected proteins, such as the DNA polymerase and the packaging adenosine triphosphatase (ATPase), are not structural proteins per se but likely copurified with the virions through association with the phage DNA. Proteins encoded by ORF1 and ORF11 were only observed with chymotrypsin cleavage; otherwise, the results were similar with both proteases. Several host proteins were also detected and assumed to be contaminants.

**Table 1.** Proteins of phage Andhra. A dash (−) indicates that there is no obvious equivalent in P68.

**Andhra ORF**	**P68 ORF**	**Amino acids**	Seq. ID. (%)*	**MS count^†^**		**Oligomer^‡^**	**Copies/ virion**	**Alias**	**Function and location^§^**
				**Tryp**	**Chym**				
1	2	90#	61.1	N.D.	3	2	6	Tip	Tail tip protein
2	3	71	32.4	2	3	–	–	–	–
3	4	169	52.4	4	6	–	–	SDB	ssDNA-binding protein
4	–	69	–	2	4	–	–	–	–
5	–	81	–	N.D.	N.D.	–	–	–	–
6	–	403#	–	61	62	2	12	Anc	RBP anchor; hydrolase
7	8	167	25.2	7	7	1	2	5TP	5′ Terminal protein
8	9	419	65.5	39	39	–	–	Pac	Encapsidation protein; packaging ATPase
9	10	763	66.2	16	22	–	–	Pol	DNA polymerase
10	11	473	68.3	18	19	2	2	LysT	Tail tip lysin (spike)
11	12	136	64.7	N.D.	5	–	–	Hol	Holin
12	13	588	69.4	138	146	6	6	Knob	Tail knob protein
13	14	285	32.3	99	99	3	15	Fib	Head fiber
14	16	299	20.1	2	3	–	–	Lys	Endolysin
15	17	609	23.5	166	166	3	36	RBP	Receptor binding protein; appendages (tail fiber)
16	18	278#	44.0	39	42	12	12	Stem	Tail stem protein (lower collar)
17	19	335#	62.1	49	53	12	12	PP	Portal protein (upper collar)
18	20	405	75.3	874	880	3/5/6^‡^	235	CP	Major capsid protein
19	21	63	60.0	8	8	1	235	CLP	Capsid lining protein (inner capsid)
20	22	107	34.0	5	5	3/6^‡^	72	Cor	Portal-proximal core protein (inner core)

*Sequence identity between equivalent proteins in Andhra and P68. Calculated using Clustal Omega.

†Normalized spectrum counts measured after either trypsin (Tryp) or chymotrypsin (Chym) cleavage. N.D., not detected.

‡In cases where the oligomeric state is ambiguous (e.g., CP), several numbers are given.

§Where the names of the equivalent proteins in Andhra and P68 diverge, the P68 name is given in parentheses.

#The sequences for ORFs 1, 6, and 16 are misannotated in the GenBank entry for phage Andhra (KY442063.1). The published sequence for ORF1 starts at nucleotide 354 (ATG), while the correct start is presumed to be at 321 (GTG), adding 11 residues to the gp1 sequence. The published sequence or ORF6 starts at nucleotide 1905 (ATG), while the true start is at 1887 (TTG), adding six residues to the gp6 sequence. The published sequence for ORF16 starts at nucleotide 15,357 (ATG), while the correct start is at 15,474 (TTG), adding 39 residues to the gp16 sequence. Similarly, the sequence for ORF17 is misannotated in the GenBank entry for phage P68 (AF513033.1). The correct start for P68 ORF17 is at nucleotide 16,096 (ATG), adding 78 residues to the N terminus of PP.

A dataset of 8818 images of Andhra virions (fig. S1A) was collected on an FEI Titan Krios electron microscope with a Gatan K3 detector, yielding a total of 230,714 particle images (Table 2). These data were subjected to three-dimensional (3D) reconstruction in RELION-3 (*34*) without any symmetry imposed (C1). 3D classification revealed that the sixfold symmetric tail existed in two orientations relative to the head, rotated by 30° to each other. Presumably, this ambiguity was due to interaction of the 6-fold tail with the 12-fold symmetric portal. The final asymmetric reconstruction of the virion therefore used only a subset of 62,061 particle images and reached 4.43-Å resolution by the Fourier shell correlation = 0.143 criterion, according to “gold standard” methods (fig. S1B).

**Table 2.** Data collection, reconstruction, and refinement data. FSC, Fourier shell correlation; RMSD, root mean square deviation.

**Data collection**					
Microscope	Titan Krios				
Voltage (kV)	300				
Detector	Gatan K3				
Pixel size (Å)	0.664				
No. movies	8818				
Frames per movie	40				
Total dose (*e*/Å^2^)	38.9				
Total no. particles	282,862				
**Reconstruction**					
	Asymmetric virion	Icosahedral capsid	Tail	Distal tail	Tail tip
Initial no. particles	230,714	186,542	159,489	159,489	159,489
Final no. particles	62,061	90,677	154,849	101,037	98,203
Symmetry	C1	I1	C6	C6	C1
Pixel size (Å)	1.33	1.33	1.33	1.33	1.33
Box size (pixels)	800^2^	512^2^	512^2^	384^2^	256^2^
Resolution (Å) FSC_0.143_	4.43	3.50	3.58	3.90	4.90
**Atomic model refinement**					
Resolution limit		3.5	3.6	4.0	
Model-to-map resolution (Å) FSC_0.5_		3.5	3.6	4.2	
No. chains		18	33	3	
No. residues		5620	5351	626	
No. non-H atoms		45,397	42,467	5116	
Map CC_box_		0.44	0.63	0.48	
Map CC_mask_		0.80	0.85	0.80	
RMSD bond length (Å)		0.003	0.004	0.003	
RMSD bond angles (°)		0.57	0.63	0.57	
Ramachandran plot					
Favored		94.7	93.0	96.3	
Allowed		5.3	7.0	3.7	
Outliers		0	0	0	
Rotamer outliers (%)		0.06	0.19	0.35	
Clashscore		6.97	12.48	9.81	

**Fig. 2.**
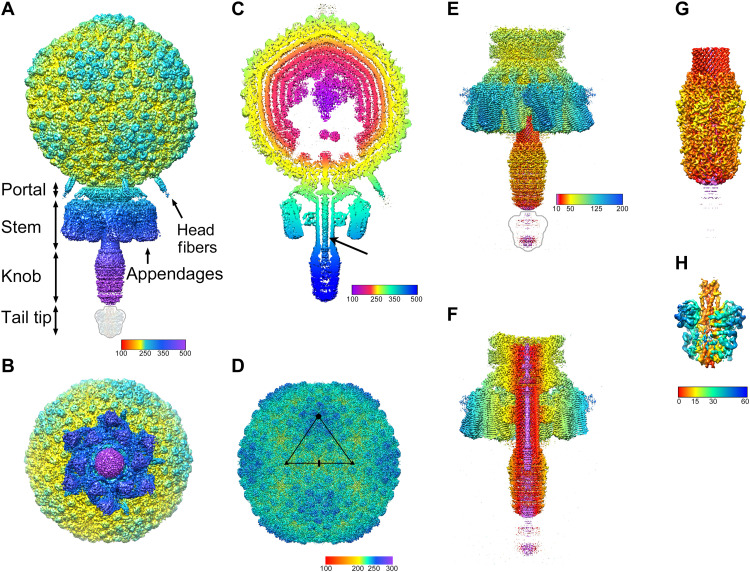
Isosurface representations of the reconstructions. (**A**) Asymmetric reconstruction of the whole virion, colored radially from the center of the capsid according to the color bar (distances in angstrom). Rendered at 5 SDs above the mean (5σ). The tail tip was not visible in the reconstruction at this cutoff and is indicated by the outline. (**B**) The asymmetric reconstruction viewed from the bottom (tail end), showing the sixfold symmetric arrangement of the appendages. (**C**) A 50-Å-thick slab through the asymmetric reconstruction, showing the concentric rings of DNA in the interior and the DNA inside the tail (arrow). Colored radially from the center of the capsid according to the color bar. (**D**) Icosahedral reconstruction of the capsid, rendered at 3σ and colored radially from the capsid center according to the color bar. The asymmetric unit triangle is shown, with fivefold, threefold, and twofold symmetry axes denoted by the pentagon, triangles, and oval, respectively. (**E**) C6 reconstruction of the tail, rendered at 3σ and colored radially from the central axis according to the color bar. The tail tip is indicated by the outline. (**F**) Cutaway view of the tail, rendered and colored as in (E). (**G**) Focused C6 reconstruction of the distal tail, showing the tail knob, rendered at 6σ and colored as in (E). (**H**) Focused asymmetric reconstruction of the tail tip, rendered at 2.5σ and colored radially from the central axis according to the color bar.

The overall structure of the Andhra virion follows that of other phages in this group, with an isometric head (capsid) around 500 Å in diameter (vertex to vertex), connected to a 400-Å-long, straight tail surrounded by 12 appendages (Fig. 2A). The head has icosahedral symmetry, with the tail attached to a portal structure located at 1 of the 12 fivefold vertices. Five trimeric head fibers are attached to the head around the portal vertex. The tail consists of a ≈150-Å-wide collar closest to the portal, followed by a ≈160-Å-long, 55-Å-wide, straight rod that widens into a 145-Å-long, 105-Å-wide knob (Fig. 2A). The 12 appendages are organized around the rod in a staggered pattern with sixfold rotational (C6) symmetry (Fig. 2B). The asymmetric reconstruction did not resolve an additional bulbous tail tip, whose structure was subsequently determined through focused reconstruction (see below).

In cross section, at least five concentric layers of DNA, spaced about 20 Å apart, are apparent inside the capsid (Fig. 2C). Toward the center of the capsid, the layers disappear and are replaced by a more disordered cone-shaped density. Immediately above the portal vertex, there is a void, reflecting a lack of consistently organized DNA in this area (Fig. 2C). There could also be minor proteins associated with the DNA, including the 5′ terminal protein and DNA binding proteins detected by MS (Table 1). A 20-Å-wide, rod-shaped density, presumably corresponding to the double-stranded DNA and the covalently attached terminal protein, extends from the capsid interior, through the portal and tail and partly into the knob.

### The capsid structure

**Fig. 3.**
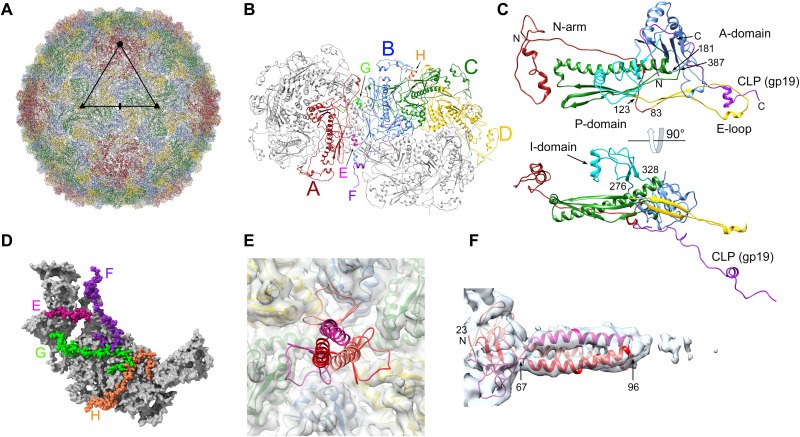
The capsid. (**A**) Ribbon diagram of the entire icosahedral capsid, viewed down a twofold axis, as in Fig. 2D. CP subunits are color coded: A, red; B, blue; C, green; D, yellow. An asymmetric unit triangle, delimited by fivefold, twofold, and two threefold axes, is shown. (**B**) Ribbon diagram showing one hexamer and one pentamer, viewed from the outside of the capsid. The four CP subunits in one asymmetric unit are colored as in (A). The corresponding CLP (gp19) subunits are color coded: E, pink; F, purple; G, light green; H, orange. (**C**) Ribbon diagram of CP subunit B, colored by domain: N-arm, red; E-loop, yellow; P-domain, green; A-domain, blue; insertion domain, cyan. The corresponding CLP (subunit F) is purple. The bottom panel is rotated 90°. Pertinent residues are numbered. (**D**) One asymmetric unit, viewed from the inside of the capsid, showing the CLPs, colored as in (B) with atoms represented as spheres. CP is shown as a gray molecular surface. (**E**) Ribbon diagram of the head fiber (gp13) trimer model (scarlet, ruby, and crimson), showing its attachment to the CP hexamer. Capsid density is shown as a transparent gray isosurface, with the corresponding CP subunit models inside. (**F**) Ribbon diagram of the head fiber trimer fitted into the asymmetric reconstruction density (transparent surface), viewed perpendicularly to the axis of the fiber and numbered by residue.

Reconstruction of the Andhra capsid with the application of icosahedral symmetry using 90,677 particles reached a resolution of 3.50 Å (Table 2, fig. S1B, and Fig. 2D), sufficient to allow atomic modeling (fig. S1, C and D). The capsid has *T* = 4 architecture and is composed of 240 copies of the major capsid protein (CP), gene product (gp) of ORF18 (gp18), arranged into 12 pentamers located on the fivefold axes and 20 hexamers located on the twofold axes (Figs. 2D and 3, A and B). (In the virion, one pentamer is replaced by the dodecameric portal at the unique vertex.) In addition, there are 240 copies of a small 63-residue protein, gp19, that we refer to as the “capsid lining protein” (CLP). There are thus four copies of CP in the icosahedral asymmetric unit, labeled A to D, with A forming the pentamers and B to D forming the hexamers, and four copies of CLP, labeled E to H (Fig. 3B).

The Andhra CP (405 residues; Table 1) has the expected HK97-like fold found in all *Caudovirales*, consisting of an N-arm (residues 1 to 82), an E-loop (83 to 122), a P-domain (123 to 180 and 344 to 386) featuring a long “spine” α helix, and an A-domain (181 to 343 and 387 to 395) (Fig. 3C). The CP A-domains form tight pentameric and hexameric clusters around the fivefold and twofold (quasi-sixfold) axes, respectively (Fig. 3, A and B, and fig. S2). The N-terminal 50 residues constitute a folded subdomain consisting of two short α helices and a loop (Fig. 3C). This domain interacts with the equivalent domain from a neighboring CP subunit in a quasi-twofold arrangement (fig. S2B). There is a 62-residue insertion (I-domain) into the A-domain (residues 276 to 328) that forms 240 prominent protrusions on the surface of the shell (Figs. 2D and 3, A and C). The I-domains are engaged in intracapsomeric contacts with the E-loop and the N-arm of two threefold-related subunits (fig. S2C). At the icosahedral threefold axes, the P-domains of three C subunits together with an α helix contributed by three D subunit E-loops come together in a tight ring (fig. S2D). At the quasi-threefold axes, the P-domains from the A, B, and D subunits, together with the E-loops of the A, B, and D subunits, form a similar structure (fig. S2, E and F).

CLP (gp19) forms an S-shaped loop that lines the inside of the capsid (Fig. 3, B to D). For subunits F to H, the N-terminal 28 residues form a hook-like structure that interacts with the A-domain of a CP subunit. The chain then passes underneath the neighboring subunit within the hexamer and forms a small α helix that connects to a CP subunit in the adjacent capsomer (Fig. 3D). For subunit E, which starts in the pentamer (CP subunit A), only residues 31 to 53, which connect with a D subunit in the hexamer, were seen (Fig. 3D). On comparing our model to the equivalent protein (gp21) from P68 (*35*), we noticed that the two proteins ran in opposite directions. We therefore reexamined the deposited P68 density (EMD-4442) and found that, when the direction of the protein was switched, it showed a better fit (fig. S3) with the fraction of atoms outside density decreasing from 30.7 to 18.7% and the model-to-map correlation increasing from 0.61 to 0.74.

In P68, gp21 was proposed to constitute a scaffolding protein (SP) (*35*)—proteins involved in capsid assembly that are found in most bacteriophages (*36*, *37*). However, on the basis of its structure and location in the capsid, we consider it more likely to be a stabilizing factor, functionally similar to the so-called “decoration proteins” in many other systems.

### The head fibers

The asymmetric reconstruction revealed head fibers attached to the five hexamers closest to the portal vertex (Fig. 2A). No fibers were seen at other hexamer positions, even when rendered at a low-density cutoff level, suggesting that they have an association with portal-related structures, as suggested for P68 (*35*). However, no such connections were observed in the Andhra reconstruction. The head fibers are not visible in the icosahedral reconstruction (Fig. 2D), both due to their low occupancy (only at 5 of the 30 hexamers) and due to the fact that they are trimers attached at icosahedral twofold axes, so that the icosahedral symmetry averaging would degrade the density.

The head fibers are trimers of the 285-residue protein gp13, which is considerably shorter in Andhra than the equivalent protein in P68 (gp14; 481 residues). The two proteins share 32% sequence identity (Table 1), mainly in the N- and C-terminal domains. The visible portion of the head fiber protein consists of a 44-residue N-terminal β-prism domain (residues 23 to 66) that interacts with the A-domains of every other CP subunit in the hexamer (Fig. 3E), followed by a 30-residue α-helical coiled coil that extends ≈55 Å from the surface of the capsid (Fig. 3F). Structure prediction with AlphaFold (*33*) and HHpred analysis (*38*) indicated that the coiled coil continues for another 105 residues, followed by a C-terminal domain that is homologous to receptor binding domains from various phages, including *Listeria* phage PSA [Protein Data Bank (PDB) ID: 6R5W] and *Lactococcus* phage TP901-1 (PDB ID: 3D8M). These structures were not visible in the reconstructions of either Andhra or P68, presumably due to high flexibility of the fiber.

### Reconstruction and modeling of the tail

To resolve the tail at higher resolution, we used focused reconstruction using a mask encompassing only the tail structures (including the portal and core proteins). An edited version of the C1 reconstruction of the virion in which the capsid density had been manually erased was used as a starting model. This reconstruction was done with C6 symmetry applied and reached a resolution of 3.58 Å (fig. S1B and Table 2). In this reconstruction, secondary structure elements and many side chains were clearly resolved in the upper (capsid proximal) part of the tail (Fig. 2E and fig. S1, E to G). The lower (distal) part of the tail was not equally well resolved in this map, however, and required separate focused reconstructions (see below).

**Fig. 4.**
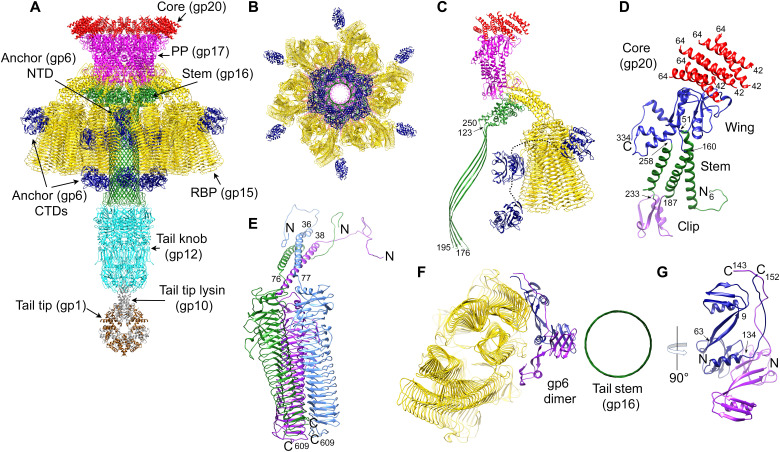
The tail structure. (**A**) Ribbon diagram of the composite model of the Andhra tail proteins, viewed from the side and colored as in Fig. 1: core protein (gp20), red; PP (gp17), magenta; tail stem protein (gp16), green; RBP (gp15), gold; RBP anchor (gp6), blue; tail knob protein (gp12), cyan; tail tip lysin (gp10), gray; tail tip protein (gp1), brown. (**B**) Model of the tail, viewed from the bottom; knob and tip not included. (**C**) One asymmetric unit of the C6 tail reconstruction, comprising 12 copies of core protein (gp20), two copies of PP (gp17), two copies of tail stem protein (gp16), six copies (two trimers) of RBP (gp15), and two copies of the anchor protein (gp6). (Knob and tip not included.) The connections between the N- and C-terminal domains of gp6 are indicated by the dotted lines. (**D**) PP monomer with the wing, stem, and clip domains colored blue, green, and purple, respectively. The corresponding six copies of the core protein (gp20) are shown in red. (**E**) One RBP trimer, with protomers colored blue, green, and purple. The N and C termini are indicated. (**F**) Ribbon diagram of a section through the tail, viewed from the bottom, showing a dimer of gp6 (blue and purple) interacting with the barrel of the tail stem (green) and the RBPs (gold). (**G**) A 90° rotated view of the gp6 dimer, viewed perpendicular to the axis of the tail. N and C termini are indicated. Pertinent residues are numbered in (C) to (E) and (G).

The C6 reconstruction was used to build atomic models for the proteins in the upper tail, including 72 copies of a small portal-proximal core protein (gp20, 107 residues; referred to as “inner core” in P68), the dodecameric portal protein (PP; gp17, 335 residues; referred to as “upper collar” in P68 and “connector” in ϕ29; see Fig. 1), the dodecameric tail stem or collar protein (gp16, 278 residues; referred to as “lower collar” in P68 and ϕ29), the 12 trimers of RBP (gp15, 609 residues; referred to as “tail fiber” in P68), and six dimers of a protein that we identified as the product of ORF6 (gp6, 403 residues; see below) (Fig. 4, A to C, and fig. S4).

### The portal, core, and tail stem proteins

The Andhra portal consists of 12 copies of PP (gp17), arranged in a 150-Å-wide ring with C12 symmetry, with a 30-Å-wide central hole (Fig. 4, A and B). There was clear density in the C6 tail reconstruction for PP from residues 6 to 334. The PP monomer consists of three domains: the wing (51 to 160 and 258 to 334), the stem (6 to 50, 161 to 186, and 234 to 257), and the clip (residues 187 to 233) (Fig. 4D). The stem domain consists of three long α helices that traverse the capsid shell. Residues 6 to 20 form a loop that interacts with the fivefold symmetric capsid shell, as previously described for P68 (*35*). The wing domain resides inside the capsid where it interacts with the portal-proximal core protein (gp20) and the DNA (Fig. 4C), while the clip domain connects with the tail stem protein (gp16).

The gp20 core protein is associated with the portal on the inside of the capsid (Fig. 4, A and C). There was clear density only for residues 42 to 64, which form a single α helix. Two bundles of three α helices sit on top of each copy of the PP, for a total of 72 copies of gp20 in the capsid (Fig. 4D). gp20 is homologous (34% sequence identity) to the somewhat longer (133 residues) inner core protein (gp22) of P68, with conserved regions interspersed by variable insertions. However, in the published structure of P68 (PDB ID: 6IAC), gp22 was modeled in the opposite direction to gp20. On the basis of its location in the genome and overall α-helical characteristic, gp20 might be equivalent to the SP (gp7) of ϕ29 (*36*, *37*, *39*). Unlike a typical SP, however, gp20 remains in the mature capsid after DNA packaging.

On the distal side of the portal, the dodecameric tail stem or collar protein, gp16, forms an elongated tube that comprises the main stem of the tail (Fig. 4, A and C). We could model gp16 from 38 residues upstream of the annotated ORF16 sequence in the GenBank entry for phage Andhra (KY442063.1), suggesting that the correct start is at nucleotide 15,474 (TTG), adding an extra 39 residues to the sequence, for a total of 278 residues (Table 1). TTG (leucine) is a common alternative start codon in *Staphylococcus*. gp16 consists of a 147-residue globular α-helical collar domain that associates with the clip domain of PP (Fig. 4, A and C). A 128-residue insertion between amino acids 122 and 250 forms a twisted β-hairpin (Fig. 4, A and C), creating a 150-Å-long tubular β barrel with an inner diameter of ≈32 Å that acts as a conduit for the DNA. The β barrel tube is ≈40 Å longer in Andhra than in P68, corresponding to an additional 28 residues in the β-hairpin. The tip of the hairpin (residues 177 to 194) forms a hook that was not visible in the tail reconstruction but was modeled in the focused reconstruction of the lower tail (see below).

### The RBPs

The 12 appendages surrounding the tail (Fig. 2, A and E) correspond to trimers of RBP, the 609-residue product of ORF15 (gp15). There are thus 36 copies of RBP in the Andhra tail, arranged in a staggered fashion (Fig. 4, A and B). The staggered C6 arrangement is unlike P68, where the 12 RBP trimers are arranged with C12 symmetry (fig. S4).

The Andhra RBP consists of an N-terminal 36- to 38-residue loop followed by a 34- to 38-residue α-helical coiled-coil stem domain, connected to a 534-residue C-terminal β helix domain (Fig. 4E). The N-terminal loop interacts with the stem and clip domains of PP and with the collar domain of gp16 (Fig. 4C and fig. S5, A and B). The C-terminal β helix domain, which comprises the majority of the RBP, consists of a stack of 19 triangular layers, each consisting of two to three β strands arranged in a regular, repeating zigzag pattern (Fig. 4E and fig. S5C). The N-terminal end of the β helix is more irregular, with longer insertions between the layers. The β helix domains of the three RBP subunits in the trimer superimpose with a root mean square deviation (RMSD) = 0.4 Å, but the N-terminal loops and coiled-coil domains diverge to accommodate the sideways attachment of the RBPs to the tail (Fig. 4E and fig. S5C). Because of the staggered arrangement of the RBP trimers, there are two types of trimers—proximal and distal—with different organization of the N-terminal domains (fig. S5D).

While the Andhra RBP is functionally equivalent to the RBP (or tail fiber) of P68 (gp17), the proteins are not homologous and have completely different structures. Whereas P68 gp17 has a structure similar to the RBP of *S. aureus* siphovirus 80α (*40*), the β helix structure of Andhra RBP is related to alginate lyases and other bacterial enzymes involved in breaking down polysaccharides. Similar proteins are found in several bacteriophages, such as the tail spikes of *Salmonella* phages Det7 (PDB ID: 6F7D) (*41*) and P22 (PDB ID: 1TSP) (*42*). Notably, the Andhra RBP is similar to the N-terminal two-thirds of the β helix domain of the ϕ29 RBP (called “preneck appendage,” gp12; PDB ID: 3GQ9) (*43*). In the reconstructions of P68 and ϕ29, the RBPs were poorly resolved, requiring separate x-ray structures to produce a pseudo-atomic structure for the tail (*35*, *44*). In contrast, the entirety of the Andhra RBP structure was well resolved. Despite the overall distinct structural folds of the Andhra and P68 RBPs, the N-terminal loops are similar (fig. S5E) and superimpose with an RMSD = 1.1 Å over 24 residues, including a stretch of 17 residues that are 100% identical between Andhra and P68 (fig. S5F). This loop represents the point of interaction between the RBPs and the portal and stem proteins, which are similar in Andhra and P68.

### The RBP anchor protein, gp6

After modeling the Andhra upper tail proteins with equivalents in P68, six well-resolved densities between the RBPs and the tail stem (gp16) remained in the tail reconstruction. Close inspection of the density revealed two tryptophan residues separated by one other residue (a WXW motif) in an α helix (fig. S1G). A sequence search revealed that this motif was found in ORF6, encoding gp6, a protein that has no equivalent in P68 (Fig. 1). Using the WXW motif as an anchor point, we were able to build the N-terminal 143 residues of gp6 into the density. Well-resolved density extended six residues upstream of the annotated start codon for ORF6 at nucleotide 1905. The true start for gp6 is thus most likely at nucleotide 1887 (TTG). gp6 forms six asymmetric dimers that bridge the RBP trimers and the tail stem protein (gp16) (Fig. 4F). The gp6 monomer consists of two α/β subdomains connected via two antiparallel β strands (Fig. 4G). In the virion, the two monomers stack on top of each other with the C termini emerging on the same side of the dimer (Fig. 4, F and G). This arrangement allows each gp6 dimer to interact with both a proximal and a distal RBP trimer (Fig. 4, C and F). Because the gp6 dimers appear to anchor the RBP trimers in place, we refer to this protein as the “RBP anchor” protein. Most likely, stabilization of the RBPs by these anchors was responsible for the well-ordered RBPs in Andhra compared to P68.

The C-terminal ≈200 residues of gp6 are predicted by HHpred to contain a hydrolase domain with homology to bacterial lipases, including the tail-associated lysin (Tal) of bacteriophage 80α (PDB ID: 6V8I) (*40*), suggesting that gp6 is involved in penetration of the cell wall. After building the gp6 N-terminal domains, there were still six unassigned, weak densities attached to the periphery of the RBPs and six densities attached at the distal end of the tail stem. These two types of densities could be superimposed with a correlation of ≈0.9, suggesting that they belonged to the same protein, and the most likely candidate was the C-terminal domain of gp6. An AlphaFold (*33*) model for the C-terminal domain of gp6 could be rigid-body fitted into both densities with high correlation (>0.85). The linkers connecting the N- and C-terminal domains could not be resolved, but on the basis of the direction of the C-terminal arms of the N-terminal domains of the two gp6 subunits, the top subunit most likely connects to the peripheral C-terminal domain, while the bottom subunit connects to the distal domain (Fig. 4C).

### The distal tail

It was clear from both the asymmetric reconstruction of the virion and the C6 tail reconstruction that the distal part of the tail was not well resolved (Fig. 2, A and E). This part corresponds to the tail “knob” and “tip” (referred to as “spike” in P68). These features were not well resolved in the P68 reconstructions (*35*) and initially appeared to be poorly ordered in Andhra as well. We presumed that this was due to an orientation ambiguity between the distal and upper portions of the tail. Although the tail knob was expected to form a hexamer with C6 symmetry, it was inconsistently aligned relative to the RBPs, with two possible orientations, differing by a 30° rotation. We used symmetry expansion and 3D classification to generate a masked, focused reconstruction of this portion with only C3 symmetry, which was then resymmetrized to C6. Refinement of the C6 reconstruction of the distal tail reached a resolution of 3.9 Å, allowing for atomic modeling (Fig. 2G, fig. S1H, and Table 2).

**Fig. 5.**
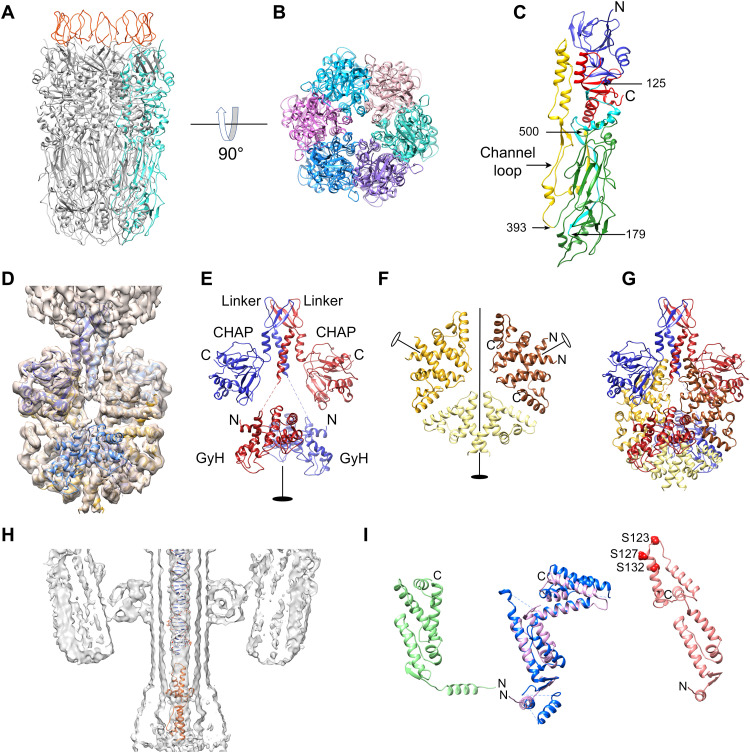
Distal tail structure. (**A**) Ribbon diagram of the tail knob (gp12) hexamer. One protomer is colored cyan. Tail stem (g16) loops are in red. (**B**) gp12 hexamer, viewed from the tail tip. Each protomer is colored in a different shade. (**C**) Ribbon diagram of a gp12 monomer: N-terminal domain, blue; N-to-C linker, cyan; tip domain, green; channel loop, yellow; C-terminal domain, red. Pertinent residues are numbered. (**D**) Isosurface (2.5σ) of the focused asymmetric reconstruction of the tail tip with gp10 and gp1 shown in blue and gold, respectively. (**E**) Ribbon diagram showing the two subunits of gp10 (red and blue). The N-terminal GyH, middle linker, and C-terminal CHAP domains are labeled, the flexible linkers are indicated by dashed lines, and the twofold symmetry axis is indicated by the filled oval. (**F**) Ribbon diagram showing the six subunits of gp1 in the tail tip arranged as three dimers, colored brown, sand, and gold. The twofold symmetry axis is indicated by the filled oval; peripheral dimer twofold axes are shown as open ovals. N and C termini are indicated on one dimer. (**G**) Ribbon diagram of the tail tip model, showing gp10 and gp1, colored as in (E) and (F) and viewed as in (E). (**H**) A 15-Å-thick slab through the asymmetric virion reconstruction, showing the central density in the tail with double stranded DNA and the gp7 model (orange) fitted in. (**I**) Left: Ribbon diagram of the AlphaFold model for gp7 (green). Middle: The modified gp7 model (pink) superimposed on the ϕ29 gp3 crystal structure (blue). Right: gp7 (salmon) modified to fit into the central tail density. N and C termini are indicated, and the serine residues near the top of the molecule (S123, S127, and S132) are shown as red spheres.

The tail knob consists of a hollow cylinder, 145 Å long and 110 Å wide, and is made up of a hexamer of gp12 (Fig. 5, A and B). gp12 has a complex, intertwined structure that is similar to those of the gp9 tail knob protein of phage ϕ29 (*45*) and the streptococcal phage C1 major tail protein gp12 (*46*), to which it could be aligned with RMSD = 1.1 Å for 212 of the 588 residues. The N-terminal part of gp12 forms a β-sandwich domain related to tail tube proteins from many members of the Siphoviridae and Myoviridae bacteriophages (*47*), connected to an elongated β sheet domain at the bottom of the hexamer via an extended connecting loop (Fig. 5C). The polypeptide chain then returns to the top of the hexamer to complete the β sheet in the N-terminal domain. Residues 393 to 500 form an extended α-helical loop (the “channel loop”) on the inside of the cylinder, which constricts the inner diameter to ≈12 Å, presumably preventing exit of the DNA and terminal proteins (Fig. 5, B and C). This loop was not resolved in the crystal structures of C1 gp12 (*46*) or ϕ29 gp9 (*45*), but the corresponding density was observed in the ϕ29 reconstruction (*44*). Most likely, these loops reorganize to become extruded through the tip of the tail during infection, enabling the resulting 12 α helices to insert into the host membrane to allow the passage of the DNA into the cell, similar to the mechanism proposed for ϕ29 (*44*). The focused reconstruction of the distal tail also resolved the hooks at the end of the β-hairpins of the tail stem protein (gp16) dodecamer that were not seen in the C6 reconstruction of the whole tail (Fig. 5A).

The distal tail reconstruction still did not resolve the bulbous density at the tip of the tail (Fig. 2, E and G). The tip density was not resolved in the P68 reconstruction either and was suggested to consist of a pentamer of gp11 (*35*), equivalent to Andhra gp10 (Fig. 1), a protein that we had previously demonstrated to act as a lysin with cell wall hydrolase activity (*29*). To resolve the tip density, we carried out another masked reconstruction focused on the tail tip without the application of symmetry, reaching a resolution of 4.9 Å (Table 2). This reconstruction revealed a twofold symmetric structure consisting mostly of α helices, with a four-helix bundle inserting into the knob (Figs. 2H and 5D).

A partial crystal structure of the N-terminal domain (residues 2 to 200) of P68 gp11 was solved previously (PDB ID: 6O43) (*48*) and was shown to be homologous to streptococcal phage C1 lysin PlyCA, for which a full-length structure was previously determined (PDB ID: 4F88) (*49*). The PlyCA structure consists of a glycosyl hydrolase (GyH) domain connected to a Cys-His aminohydrolase/peptidase (CHAP) domain. In phage C1, these enzymatic activities are involved in breaking down the streptococcal cell wall. An AlphaFold model for Andhra gp10 revealed the two expected domains (GyH and CHAP), separated by a helix-sheet-helix linker domain (residues 211 to 277). The three domains were individually fitted into the tail tip reconstruction, revealing a gp10 dimer with the N-terminal GyH domains at the bottom of the tip and the C-terminal CHAP domains at the top (Fig. 5, D and E). The two α helices of the linker domain fit into the connecting densities between the tip and the knob, with the β sheet penetrating into the gp12 knob protein above (Fig. 5, D and E). The density corresponding to the N-terminal domain was weak (Fig. 5D), suggesting low occupancy and high degree of flexibility.

After fitting the gp10 dimer into the tip density, it was clear that the tip contained at least one other, primarily α-helical protein. After subtracting the gp10 density, the remaining density appeared as a flat disc with quasi-threefold symmetry. Closer analysis revealed that the disc was made of three dimers, with the twofold symmetry axis of the tip passing through one dimer and the other two dimers on opposite sides of the tip (Fig. 5, D, F and G). The shape and size of the density were consistent with a mostly α-helical protein of approximately 90 residues. The only unassigned candidate proteins of the right size were gp1 (90 residues), gp2 (71 residues), gp4 (69 residues), and gp5 (81 residues). We generated AlphaFold models for each of these proteins. gp2, gp4, and gp5 were a poor match for the tail tip density, but gp1 had a compact, α-helical structure that could be fitted as three dimers into the tail tip disc density (Fig. 5, D to F). In particular, the C-terminal helix had several aromatic residues that matched bulky side chain densities in the map. We noticed that the predicted N-terminal α helix was too short for the density. Upon reinspecting the sequence, we realized that the published sequence was missing 11 residues, with the most likely start being a GTG (valine) codon upstream of the annotated sequence in GenBank (KY442063.1). Gp1 has homologs in other staphylococcal picoviruses, including gp2 from P68, but no other homologs that could give an indication of its function, such as an enzymatic activity.

### Internal tail density

On the inside of the tail, a ≈22-Å-wide, elongated density stretched from the capsid, through the portal and tail tube to the knob, where it widened slightly (Fig. 2, C and F). In the collar at the interface between the portal and tail tube, the density formed a disc-like shape. The central density was not well resolved in either the C6 tail reconstruction or the asymmetric reconstruction of the virion and could not be modeled. Attempts at masked and focused reconstruction without symmetry were not successful. In the P68 reconstruction, the internal density was proposed to correspond to a trimer of P68 gp8 (equivalent to gp7 in Andhra) and designated as “needle” based on its high α-helical content and elongated shape (*35*), by analogy with the trimeric needle protein (gp26; PDB ID: 4ZKU) of phage P22 (*50*). However, this similarity is most likely only superficial, because these two systems are highly dissimilar, and the P22 needle is located on the outside of the P22 tail tip.

Instead, the internal density most likely corresponds to one end of the genome. The width of the density matches that of double-stranded DNA (Fig. 5H). The bulbous density inside the knob presumably corresponds to the covalently attached Andhra terminal protein (5TP). On the basis of comparisons with the ϕ29 genome, and excluding ORFs with known functions, the most likely candidate for the Andhra 5TP is gp7 (Fig. 1). An AlphaFold model of gp7 has an elongated, α-helical structure with the same topology as the ϕ29 terminal protein, gp3, for which a crystal structure bound to the ϕ29 DNA polymerase was previously determined (PDB ID: 2EX3; Fig. 5I) (*51*). The two proteins superimpose with an RMSD of 2.2 Å after rotating the N- and C-terminal subdomains of gp7 (Fig. 5I). Fitting of gp3 into the ϕ29 reconstruction required extensive modification (*44*), suggesting that the protein takes a different, more elongated conformation in the virion compared to the polymerase-bound form. When gp7 is modified similarly (Fig. 5I), it fits roughly into the bulbous density inside the knob (Fig. 5G). In ϕ29, the 5′ end of the DNA is covalently attached to residue S232 of gp3. It is not known which residue is bound to the DNA in Andhra, but in this hypothetical gp7 model, several serine residues, including S123, S127, and S132, are in a position where they could interact with the DNA (Fig. 5I).

## DISCUSSION

All tailed bacteriophages are considered to belong to the same lineage (the *Caudovirales*), which is evident from similarities in the structures of many key proteins, including the major CP, the PP, and the major tail proteins (*52*). Traditionally, the *Caudovirales* are divided into three families based on the structure of the tail: long and contractile (*Myoviridae*), long and flexuous (*Siphoviridae*), or short (*Podoviridae*). Picoviruses like Andhra, P68, and ϕ29 have been considered a subfamily (*Picovirinae*) in the *Podoviridae* family due to the presence of a short tail (*22*). However, these phages have many features that distinguish them from other podoviruses, like P22 and T7, including their small genomes and their distinct replication and DNA packaging strategy (*28*). The International Committee on Taxonomy of Viruses recently separated out the picoviruses into three new families (*23*, *24*): *Salasmaviridae* [which includes *Bacillus* phage ϕ29 and related viruses like B103 and GA-1 (*26*)], *Guelinviridae*, and *Rountreeviridae* [the latter including Andhra, P68, and 44AHJD (*27*)]. While the genomic organization differs somewhat between the Salasmaviridae and the Rountreeviridae, they are clearly related (Fig. 1). The 3D structures of key proteins are also generally conserved between the two families, with some exceptions where host interactions are involved, e.g., the RBPs. In a fascinating twist to this genealogy, structures of the RBPs of Andhra and P68 show close relationships to phages belonging to divergent lineages: The P68 RBP is highly similar to the RBP of staphylococcal siphovirus 80α (*40*), while the Andhra RBP resembles proteins from phages that infect a variety of hosts, including *Bacillus* (ϕ29) and *Salmonella* (Det7). These relationships are a reflection of the extensive horizontal evolution between phages from vastly different lineages.

Assembly of capsids of the *Caudovirales* bacteriophages generally requires the assistance of a SP, which acts catalytically on the CP to ensure correct assembly (*36*, *37*). [One exception are the HK97-like phages, such as *S. aureus* phage ϕ12, in which an N-terminal scaffolding domain of the CP serves the same purpose (*53*, *54*).] ϕ29 has a SP, gp7, that appears to be similar to those of other phages, including staphylococcal phage 80α and its satellite SaPI1 (*39*, *55*, *56*); however, its location in the ϕ29 capsid is still uncertain (*44*).

In contrast, Andhra and P68 do not have a direct equivalent to the ϕ29 SP. Structurally, the Andhra protein most similar to a classical SP is the portal-proximal core protein, gp20, which, like other SPs, is predominantly α-helical. The location of ORF20 in the Andhra genome is equivalent to that of the SP gene (ORF7) in ϕ29: at the beginning of the structural operon, preceding the CP gene (Fig. 1). Unlike ϕ29, Andhra and P68 have an additional ORF between that of the core protein and the CP, which was interpreted as the SP gene in P68 (*35*). However, this protein—the CLP—does not resemble a SP and more likely serves a stabilizing role. The C termini of the core proteins form α-helical bundles that interact primarily with the portal (Fig. 4, C and D). In P68, the N terminus of gp21 was shown to interact with CP (*35*), as expected for a SP, but we did not observe density for this part in Andhra. There is a precedent for a portal-proximal location of the SP in ϕ29, where gp7 was found to bind to the gp10 PP and probably provides a bridge between the portal and the capsid (*57*, *58*). A key role of SPs may be to ensure incorporation of the portal during nucleation of capsid assembly. Unlike a typical SP, however, the core protein remains associated with the capsid in the mature virion (*36*, *37*). Perhaps the portal-proximal organization that we observe in Andhra and P68 is only the subset of the scaffolding that remains in the capsid.

From these observations, it is still uncertain if Andhra and its relatives use a catalytic SP during assembly or if the process is somewhat different from other Caudovirales phages. Picoviruses do not undergo the large expansion and major structural transitions associated with capsid maturation and DNA packaging observed in other tailed phages. It is also worth noting that ϕ29 differs from Andhra and P68 in having a prolate, rather than an isometric capsid. Further experiments are needed to ascertain whether the portal-proximal core protein is a SP.

Unlike most *Caudovirales* phages, which package concatemeric DNA, picoviruses use linear, monomeric DNA substrates with a terminal protein (5TP) bound to each 5′ end (*25*, *59*). The terminal protein gets packaged along with the DNA and is ejected with the DNA during infection. Packaging is carried out by a “packaging motor” ATPase (gp8 in Andhra). The 5TP is also responsible for recruitment of the DNA polymerase and initiation of replication (*25*, *28*). In ϕ29, the gene encoding 5TP (ORF3) immediately precedes the DNA polymerase gene (Fig. 1). Vybiral *et al.* (*27*) suggested that the P68 terminal protein was gp11 (equivalent to Andhra gp10), but gp10 encodes a lysin (*29*) that we have identified as part of the tail tip (Fig. 5). On the basis of structural analysis, the most likely candidate for the Andhra 5TP is gp7 (Fig. 5I). Density observed inside the tail knob thus most likely corresponds to this protein (Fig. 5H).

Because WTA is often the primary receptor for staphylococcal phages, the type of WTA present on the surface of the host is a key host range determinant (*60*–*62*). Most strains of *S. aureus* has WTA based on a phospho-glycerol (GroP) polymer backbone, whereas *S. epidermidis* and other coagulase-negative staphylococci have WTA based on a phospho-ribitol (RboP) backbone (*32*, *63*, *64*). In both cases, this backbone is derivatized by various sugars and amino acids. We previously isolated several picoviruses with specificity for either *S. aureus* or *S. epidermidis* (*29*, *30*). We analyzed the sequences of RBPs from several of these and show that they fall into two distinct groups, commensurate with the type of WTA of their hosts: Those that infect *S. aureus* have RBPs similar to that of P68, while those that infect *S. epidermidis* have RBPs similar to that of Andhra (Table 3). The P68 RBP is closely related to that of the *S. aureus* infecting siphovirus 80α (*40*), suggesting that the RBP structure in general follows the host rather than the lineage of the phage. The slight homology between the two groups corresponds to the conserved N-terminal sequence, where the proteins interact with the more conserved portal and stem proteins (fig. S5, E and F). The head fibers are probably also involved in host interactions. The C-terminal domain of gp13 is related to receptor binding domains from several phages that infect a variety of hosts, suggesting that it might bind to alternate surface structures that are common to multiple hosts.

**Table 3.** Sequence identity between the RBPs of various picoviruses. Percentage identity was calculated in Clustal Omega. The *S. epidermidis*-infecting phages Andhra, Pontiff and JBug18 and the *S. aureus*-infecting phages Pabna, 44AHJD and P68 form separate clusters. GenBank IDs are as follows: Andhra, KY442063.1; Pontiff, MH972262.1; Jbug18, MH972263.1; Pabna, MH972260.1; 44AHJD, AF513032.1; P68, AF513033.1.

	**Andhra**	**Pontiff**	**JBug18**	**Pabna**	**44AHJD**	**P68**
Andhra	100.00					
Pontiff	97.37	100.00				
JBug18	97.04	99.34	100.00			
Pabna	23.45	23.45	23.24	100.00		
44AHJD	23.03	23.03	22.81	95.66	100.00	
P68	23.03	23.03	22.81	95.66	100.00	100.00

Binding to WTA is only the first step in the infection mechanism. Enzymatic activities associated with the tail are then needed to degrade the cell wall. Several tail proteins have enzymatic activities: gp6, a predicted hydrolase/lipase; gp10, which has a GyH/peptidase domain connected to a CHAP domain (*29*); and RBP, with predicted homology to alginate lyases. [Andhra gp14 is also a lysin with amidase activity but is not associated with the tail and is assumed to act from within during cell lysis (*29*).] It is likely that conformational changes occurring upon the initial binding of the RBPs to WTA release the lytic activities associated with gp6 and gp10 to break down the cell wall. No equivalent of gp6 exists in P68, suggesting that the associated enzymatic activity may be specific to *S. epidermidis*. The role of gp1 in the tail tip is not clear but is most likely also involved in penetration of the cell wall. It is not known if additional, specific interactions between the tail and membrane-associated proteins in the host cell are required. Penetration of the membrane might cause the tail tip to fall apart, allowing the α helices of gp10 to disengage from the tail knob, leading to a conformational change in the internal α helices in the knob, similar to that described for ϕ29 (*45*). Opening up of the tail tube would then allow the terminal protein to escape, pulling the DNA along with it, similar to the process proposed for the tape measure protein of phage 80α (*40*).

Most phages now in use for phage therapy are large, lytic myoviruses that have genomes of >150,000 base pairs that encode hundreds of genes, most with unclear functions (*20*). Furthermore, phage therapy typically uses cocktails of multiple phages. In contrast, picoviruses are attractive candidates for therapeutic use due to their small and well-defined genomes, lack of virulence factor genes, strictly lytic life cycle, and unique DNA packaging strategy that precludes generalized transduction. However, staphylococcal picoviruses are typically restricted to a narrow range of hosts. This narrow host range is a double-edged sword: On one hand, it allows the phages to target only the specific pathogen causing the disease; on the other hand, it means that the phage may need to be tailored to the patient in each specific case. Genetic manipulation of lytic picoviruses like P68 and Andhra has recently been facilitated by CRISPR technology (*65*), making possible the production of custom-made phages tailored to a specific purpose. With the present study, we have gained a better understanding of the structures and functions of the Andhra gene products and the determinants of host specificity, paving the way for a more rational design of custom phages for therapeutic applications. A better understanding of host range and the infection process will facilitate the development of this group of phages for therapeutic applications.

## MATERIALS AND METHODS

### Purification and imaging of phage Andhra

Andhra was originally isolated from raw sewage and purified as previously described (*29*), yielding a lysate (≈1× 10^9^ plaque-forming units/ml) of which 150 μl was combined with 300 μl of an overnight culture of *S. epidermidis* RP62a, 7 ml of semisolid “sloppy” agar (2.3 g of brain heart infusion and 0.75 g of agar in 250 ml of water), and 7 μl of 5 M CaCl_2_ at 55°C. The mixture was plated onto tryptic soy agar with 5 mM CaCl_2_ and allowed to solidify before incubating at 37°C overnight. Complete lysis occurred, and the sloppy agar layer containing phage was collected and combined with 20 ml of tryptic soy broth per plate. Samples were then vortexed and centrifuged at 4400*g* for 5 min. The supernatant was transferred to a new tube and centrifuged at 4400*g* for 5 min. After the second centrifugation, all lysates were combined and filtered via vacuum filtration. The clarified lysates were made up to 10% PEG 8000 and 1.0 M NaCl and incubated overnight at 4°C. The solutions were centrifuged at 11,000*g* for 45 min, and the supernatants were discarded. The pellet containing Andhra was resuspended in 2 ml of phage buffer [50 mM tris-HCl (pH 7.8), 100 mM NaCl, 1 mM MgSO_4_, and 4 mM CaCl_2_] by gentle shaking. Deoxyribonuclease I (10 mg/ml) and ribonuclease A (2 mg/ml) were added to the pellet before incubating at 37°C for 30 min with agitation. The sample was collected and layered onto a CsCl step gradient with densities of 1.3, 1.5, and 1.7 g/ml and centrifuged in a SW41 rotor at 140,000*g* for 4 hours at 12°C. Visible bands were removed from the tubes and centrifuged separately in a Ti70 rotor at 185,000*g*, 4°C for 1 hour. The supernatant was removed, and pellets were resuspended in 100 μl of phage buffer at 4°C overnight with gentle shaking. The concentrated phage was then dialyzed in phage dialysis buffer [20 mM tris-HCl (pH 7.8), 50 mM NaCl, 1 mM MgSO_4_, and 4 mM CaCl_2_].

### Mass spectrometry

Andhra in phage buffer was prepared in Novex NuPage LDS sample buffer (Invitrogen), separated on a Novex NuPage 10% bis-tris polyacrylamide gel (Invitrogen), and stained overnight with Novex Colloidal Blue (Invitrogen). One entire lane was divided into four fractions, which were equilibrated in 100 mM ammonium bicarbonate and digested overnight with trypsin (20 μg/ml; Trypsin Gold, MS grade; Promega) or chymotrypsin (20 μg/ml) before liquid chromatography–MS analysis. The peptide digests were separated by high-performance liquid chromatography on a C-18 reverse phase column using a 0 to 85% acetonitrile gradient with 0.1% formic acid, running in-line with a Thermo Q Exactive HFx mass spectrometer. The mass data were searched with SEQUEST using a database containing the predicted Andhra ORFs (GenBank ID KY442063.1) and the *S. epidermidis* RP62a sequence (GenBank ID CP000029). The list of peptides was filtered using Scaffold 5.1.2 (Protein Sciences, Portland, OR) with peptide and protein thresholds of 80 and 95%, respectively.

### Cryo-EM sample preparation and data collection

Purified Andhra virions in phage dialysis buffer were applied to nickel Quantifoil R2/2 grids and plunge-frozen using an FEI Vitrobot Mark IV at 100% humidity with 5-s blot time and blotting force 5. Grids were screened and a preliminary dataset consisting of 110 movies was collected using SerialEM on an FEI Tecnai F20 microscope operated at 200 kV, equipped with a Gatan K3 detector, with 1.45-Å pixel size. Each movie consisted of 20 frames and a total electron dose of 32 *e*^−^/Å^2^. Frame alignment was done on the fly using the built-in SerialEM module. A dataset consisting of 8818 movies were collected with a FEI Titan Krios microscope at Purdue University’s Midwestern Center for Cryo-Electron Microscopy, operated at 300 kV using a Gatan K3 electron detector with a pixel size of 0.664 Å in superresolution mode, with nominal defocus range of 1.0 to 3.5 μm. Each movie contained 40 frames and used a total electron dose of 39 *e*^−^/Å^2^ (Table 2). Frame alignment was done with MotionCor2.

### 3D reconstruction

3D reconstruction was done predominantly using RELION-3, v.3.0.8 and v.3.1 (*34*). A dataset consisting of 5216 particles was picked semiautomatically from the F20 images and reduced to 4348 particles after 2D classification. A C1 starting model was made ab initio in RELION. The final reconstruction from this data reached 10.6-Å resolution. For the Titan Krios data, a total of 282,862 particles were picked semiautomatically in RELION (Table 2). 2D classification yielded a dataset of 230,714 particles with an appearance consistent with Andhra virions. The C1 reconstruction from the F20 data was used as a starting model for 3D classification and refinement. The final C1 reconstruction used a subset of 62,061 particles and reached 4.43-Å resolution. For icosahedral reconstruction of the capsid, the images were reextracted to be centered on the capsid. A starting model was made by icosahedrally averaging the asymmetric reconstruction after removing the tail density. After 3D classification, the best 90,677 particles were subjected to reconstruction with icosahedral (I1) symmetry, yielding a final map at 3.50-Å resolution. For reconstruction of the tail, images were reextracted with a center approximately in the middle of the tail as seen in the asymmetric reconstruction and with a larger box size to accommodate the length of the tail. As a starting model, the capsid density was manually erased from the asymmetric reconstruction using UCSF Chimera (*66*), and the tail density was centered in the map. The best 154,849 particles were used for 3D refinement with C6 symmetry, yielding a final map at 3.58-Å resolution. It was clear from this map that the distal part of the tail, including the knob and tail tip, was not well resolved, presumably due to ambiguity with the 12-fold symmetry of the tail stem. The data were therefore symmetry expanded to C12 using the relion_particle_symmetry_expand script, followed by masked 3D classification focused on only the distal tail. Reconstruction of the distal tail with C6 symmetry reached a resolution of 3.90 Å. This map still did not resolve the tail tip, so another focused, masked reconstruction was carried out by C6 symmetry expansion and reconstruction without the application of symmetry, reaching a resolution of 4.90 Å (Table 2).

### Atomic model building

All maps were sharpened using the PostProcess function in RELION and the known MTF of the K3 detector before model building. For most of the Andhra proteins, model building was done de novo using Coot (*67*). In some cases, starting models were generated in I-TASSER (*68*) or using AlphaFold (*33*) from within ChimeraX (*69*) or using a homologous protein. Where the density did not allow de novo modeling, models were rigid-body fitted into the density using UCSF Chimera (*66*) followed by manual editing and real-space refinement in Coot. For the high-resolution maps of the icosahedral capsid, C6 tail, and knob, local real-space model refinement was done in Coot, followed by global real-space and B-factor refinement in Phenix (*70*). For the tail, refinement included 33 protein chains, while capsid refinement included 18 protein chains (one asymmetric unit plus surrounding subunits) (Table 2). All models were checked in MolProbity (*71*).

## Notes

View/request a protocol for this paper from *Bio-protocol*.
